# Spatial variation of short birth intervals and their determinant factors among reproductive women in Ethiopia using a geographically weighted regression model

**DOI:** 10.3389/fmed.2024.1363844

**Published:** 2024-07-09

**Authors:** Gezachew Gebeyehu Arega, Aweke Abebaw Mitku, Nuru Mohammed Hussen, Shegaw Mamaru Awoke, Haymanot Berelie Berehan, Kasaneh Jigar Alem

**Affiliations:** ^1^Department of Statistics, Samara University, Semera, Ethiopia; ^2^Department of Statistics, Bahir Dar University, Bahir Dar, Ethiopia; ^3^Department of Statistics, Assosa University, Assosa, Ethiopia; ^4^Department of Statistics, Jijiga University, Jijiga, Ethiopia

**Keywords:** short birth interval (SBI), CSA, EAS, EMDHS, OLS, geographically weighted regression (GWR)

## Abstract

**Background:**

In low- and middle-income nations, a significant proportion of maternal and infant deaths are caused by a short birth interval (SBI). In Ethiopia, it is the main factor contributing to maternal and infant mortality. Understanding the spatial distribution of SBIs, i.e., birth intervals of less than 33 months, and the factors that influence them is important for categorizing and promoting targeted interventions. This study used a geographically weighted regression model to evaluate the factors associated with SBIs in hot areas of Ethiopia.

**Methods:**

The 2019 Ethiopian Mini Demographic and Health Survey, which is nationally representative, provided the data for this study. The first step in the two-stage cluster design used to collect the data was enumeration areas, and the second stage was households. The survey was conducted between 21 March 2019 and 28 June 2019. A hot spot analysis (local Getis-Ord Gi* statistics) was initially used to investigate spatial variation in SBIs. Geographically weighted regression was used to examine the regional variation in the relationship between SBIs and the factors that cause them.

**Result:**

The study indicated that the overall proportion of SBIs among women in Ethiopia was 43.2%. The values for Global Moran’s I (Moran’s *I* = 0.773 and *p* < 0.001) showed the presence of significant SBIs clustering in Ethiopian administrative zones in Ethiopia. High-risk areas of the SBIs include Jarar, Doolo, Shabelle, Afder, Liben, Korahe, Nogob, West Harerge, Guji, Sidama, and Assosa zones.

**Conclusion:**

Living in a geographic region with a high proportion of uneducated women, women lacking breastfeeding practices, and followers of Orthodox religions increased the proportion of SBIs. Our full map of hot spots for short birth spacing and the factors that affect them helps in the implementation of precise public health measures for decision-makers.

## Introduction

The period between the birth of the child being studied (the index child) and the birth that occurred right before it is known as the short birth interval (SBI). Given the implications for fertility, maternal health, and child health, SBIs have drawn more attention in studies on demography and public health (1). The majority of health risks are related to SBIs between pregnancies. There are health risks due to both widely spaced and closely spaced pregnancies (2). Poorly spaced pregnancies have been linked to poor mother and child health outcomes across the world. Every year, 11 million children under the age of 5 are believed to have died naturally, with poor nations accounting for 99% of those deaths (3).

Globally, a birth interval of less than 18 months is associated with a higher risk of maternal, newborn, under-5, and neonatal mortality (3). According to data from 18 developing countries (Africa, Asia, Latin America, and the Middle East) and a global comparison study of 77 countries using Demographic and Health Survey (DHS) data, a birth interval of 3 years or more improves the survival status of mothers, children under 5, and infants (4). Ethiopia is the second most populated country in Africa, with a population of over 100 million and a fertility rate of 4.6 children per woman. Due to sociocultural and religious factors, it has not experienced much progress in reducing births, like many other African nations (5). Due to sociocultural and religious issues, Ethiopia has, like many other African nations, shown little change in the reduction of fertility thus far (6). Early first marriages, the desire for more children, and poor contraceptive use due to religious beliefs all have an impact on fertility. In Ethiopia, SBIs have an impact on neonatal, infant, and childhood mortality rates in addition to fertility (7).

A 2016 Ethiopian DHS found that extending the birth interval by at least 2 years reduces infant mortality by 50% and fertility by 43% (8). Greater benefits come from spacing out successive pregnancies and abortions, as well as by lowering the risk of unsafe abortion-related complications. Additionally, it promotes children’s development by improving the previous child’s nutritional status (8). Numerous research used various variable sets and statistical techniques, such as survival analysis, logistic regression, and linear regression models, to examine the factors influencing fertility in Ethiopia (9, 10).

The binary logistic regression model and an auto-logistic regression model are used for identifying the relation between SBIs and explanatory variables, as well as to model spatial data, with spatial effects accounting for spatial dependence (9). For instance, regression models that violate the assumptions of independence and normality for errors with a constant variance are handled poorly by all the above classic statistical models and methods (11).

For the study of spatial data, several studies have limited the use of regression models using geographical factors. Additionally, investigations carried out in Ethiopia were unable to identify the factors associated with SBIs in the mini-EDHS 2019. The current study aimed to investigate the spatial variation of the SBIs and its determinant factors among reproductive women in Ethiopia using a geographically weighted regression (GWR) model.

## Method

### Study area

**Figure 1:**
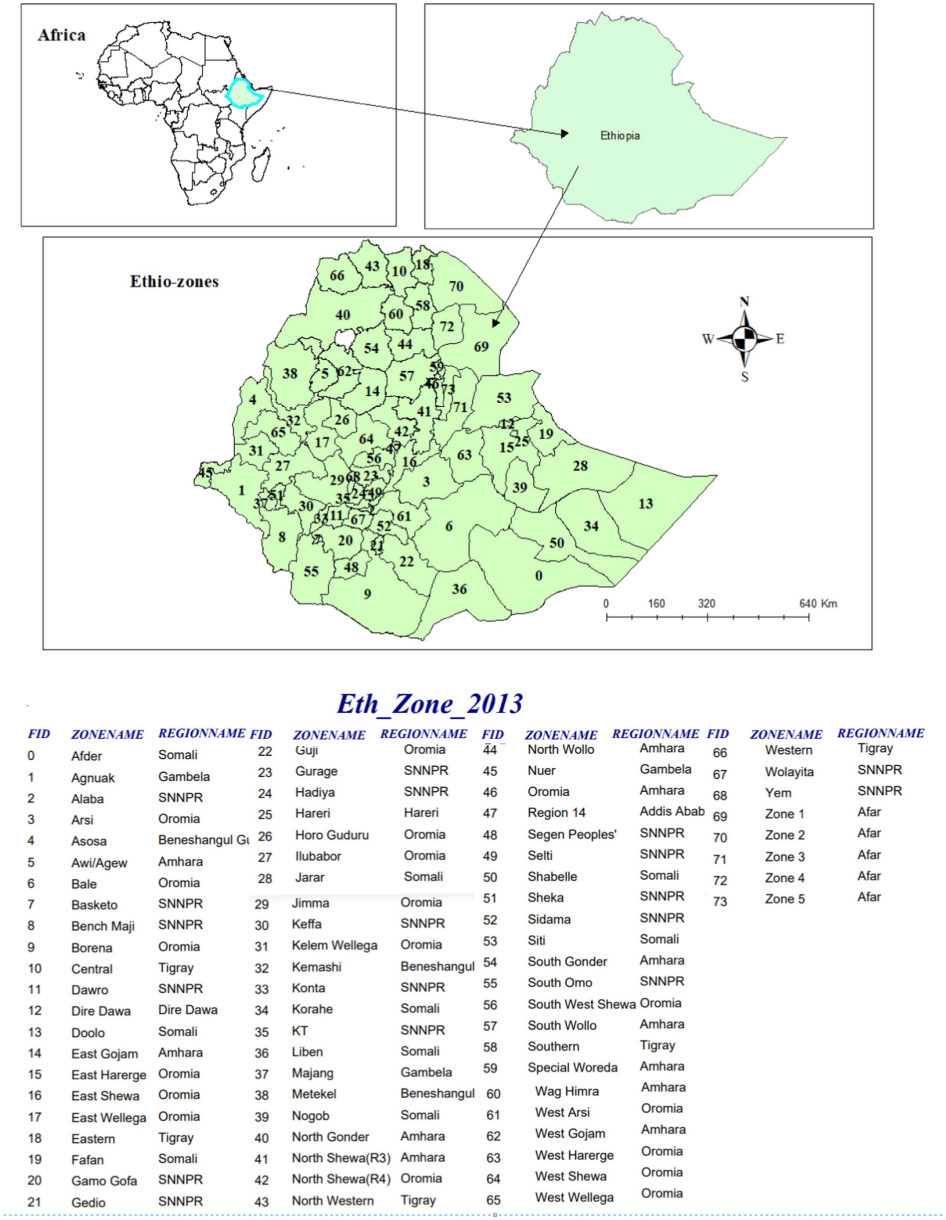
Area map of Ethiopia with 74 administrative zones.

The study was carried out in Ethiopia, which is located in the Horn of Africa. The country covers 1.1 million km^2^ on its high central plateau, which reaches as low as 110 m in the Afar valley and as high as 4,550 m above sea level. There are two administrative cities, Addis Ababa and Dire Dawa, and nine regional states in the country: Tigray, Afar, Amhara, Oromia, Somalia, Benishangul Gumuz, Southern Nations, Nationalities, and People’s Region (SNNPR), Gambella, and Harari. The regions are further divided into 74 administrative zones (see Figure 1) (12).

### Study design, period, and setting

From 21 March to 28 June 2019, a cross-sectional survey based on the community was conducted. EAs were used as the sample units for the first stage of the stratified, two-stage cluster design used to choose the 2019 mini-EDHS sample, while households served as the sampling units for the second stage. The complete mini-EDHS report includes a detailed description of the sampling process.

### Data source and study population

All Ethiopian women in the reproductive age range (15–49) were part of the source population. The study population consisted of women of reproductive age who had given birth within the 5 years before the survey.

### Sample size and sampling procedure

The analysis used a weighted sample size of 4,793 people who had given birth within the recent year. To improve the representativeness of the sample data, weighted values were used. Participants were selected through a stratified, two-stage cluster sampling process. The DHS website provided access to each mini-EDHS report, which included a thorough sampling technique.^1^

### Data collection tools and procedures

When the request for this study was made and the www.dhsprogram.com website was visited, the DHS Program provided the data. Data from the Ethiopian DHS were gathered using the stratified two-stage sampling method.

## Variables

### Outcome variable

The study’s outcome variable was the SBIs of women. The outcome variable was binary, with 1 indicating an SBI for women and 0 indicating not. Finally, for spatial analysis, including spatial regression analysis, the weighted proportion of women’s SBIs per EAs, a continuous variable, was used.

### Independent variable

The potential explanatory factors used in the current study’s exploratory regression were women’s education level, wealth index, marital status, religion, survival status of the index child, breastfeeding status, contraceptive utilization, and type of residence.

### Data management and analysis

SPSS version 26 and STATA version 16 are the software used to manage the data. We used multiple imputation methods for handling missing data in this study. These methods can account for the complex structure of the data and the relationships between variables while generating multiple imputed datasets to capture uncertainty. Additionally, robust statistical methods were used to handle outliers, ensuring that extreme values do not unduly influence the analysis results. Sample weighting was performed before further analysis, and the spatial analysis was performed using Arc GIS. Before conducting the spatial analysis, each predictor variable was expressed as binary, and after calculating the weighted proportions of the candidate predictor variables and SBIs (the outcome variable) for each enumeration area, ArcGIS version 10.8 was uploaded. Then, global ordinary least squares (OLS) linear regression was performed to model the outcome variable (SBIs) in terms of its relationships to a set of predictor variables. All of the OLS assumptions and diagnoses required by this method were thoroughly assessed and checked. The OLS summary report and diagnostic output tables provided the model summary diagnostics. The corrected Akaike Information Criterion (AIC), the coefficient of determination, the Joint F statistic, the Wald statistic, Koenker’s Breusch–Pagan statistic, and the Jarque–Bera statistic are all included in the diagnostics for both. After the OLS assumptions and diagnosis were satisfied, the GWR analysis was then used to model the spatially varying relationships between the SBIs and the candidate predictor variables. Finally, the coefficients of the significantly associated predictor variables were mapped to determine which predictor contributed the greatest influence on the proportion of SBIs across the different geographical locations of Ethiopia.

### Spatial autocorrelation

We used Arc GIS 10.8 to identify hot spot zones and perform spatial autocorrelation. The spatial autocorrelation measurement (Global Moran’s I) was applied in Ethiopia to determine if SBIs were randomly distributed, clustered, or dispersed. Women’s SBIs are spread when the Moran’s I value is close to −1, clustered when it is close to +1, and randomly distributed when it is zero (13).

### Hot spot analysis

The hot spot analysis was conducted using the proportion of SBIs in each administrative zone of Ethiopia as input. The features with either hot spot or cold spot values are revealed by the Hot Spot Analysis (Getis-Ord Gi* statistic) of the z-scores and significant *p*-values (14). A hot spot refers to the occurrence of a high proportion of SBIs that would be clustered together on the map, whereas a cold spot refers to the occurrence of women who have a low proportion of SBIs that would be clustered together on the map.

### Associated factors of the short birth interval of women

Women are nested within a cluster in the EDHS data, and we estimate that these women in the same cluster will be more similar to one another than women across the country. This violates the equal variance across clusters and observation independence requirements of the standard regression model (1, 15). This suggests that the advanced GWR model must be used to account for cluster heterogeneity (16).

### Ordinary least squares regression

Geographical regression modeling was used to identify indicators of the observed geographical clustering of SBIs when the hot spot zones were identified. It was determined to fit an OLS regression first. The accuracy of the OLS regression results depends on the regression model satisfying all the criteria allowed by this approach. A well-stated OLS model should have a statistically significant coefficient of predictor variables with a positive or negative sign. Furthermore, there should not be any multicollinearity that is not a correlation between the predictor variables. The model should be unbiased (heteroscedasticity or non-stationarity). The residuals should be normally distributed and reveal no spatial patterns. The model should include important predictor variables. The residuals must be free from spatial autocorrelation (17). These assumptions were also checked appropriately. The OLS regression equation (18) is presented as:


$$Y_{i}=\beta +\overset{P}{\underset{K=1}{\sum }}\left(\beta_{k}X_{ik}\right)+\epsilon_{i}$$

where i=1,2,…n;β0,β1,β2,…βp are the model parameters, yi is the outcome variable for observation i, $X_{ik}$ are explanatory variables and ${\;}_{1},\epsilon_{2},\ldots \epsilon_{n}$ are the error term/ residuals with zero mean and homogenous variance σ2 . Models with high adjusted R2 values are found by exploratory regression, which helps find a model that satisfies the OLS method’s basis. Additionally, it finds models that satisfy all of the OLS method’s assumptions (19).

## Results

Table 1 presents the weighted proportions of SBIs among candidates based on sociodemographic and obstetric variables. The overall proportion of SBIs was 43.2% in this study. Of all respondents who have SBIs, 1,569 (75.8%) were observed from rural areas, and only 500 (24.5%) were observed from urban areas. Approximately 600 (29%) SBIs were observed among orthodox religious followers. Similarly, among women who had SBIs, 948 (45.8%), 414 (20%), and 707 (34.2%) were from poor, middle, and rich wealth households, respectively. The majority of women who had SBIs were married women. Likewise, the majority of women who had SBIs also did not use contraceptive methods. In general, the proportion of SBIs decreases as the education level increases.

**Table 1:** Association of sociodemographic and obstetric characteristics of women with a short birth interval (SBI) in Ethiopia, MEDHS.

Variable	Category	Weighted frequencies	Short birth interval (SBI)	Non-short birth interval	*p*-value
Residence	Rural	6,024 (67.8%)	1,569 (75.8%)	1,972 (72.4%)	0.007
	Urban	2,861 (32.2)	500 (24.5%)	752 (27.6%)	
Education level	No education	3,589 (40.4%)	1,351 (65.3%)	1,633 (59.9%)	<0.001
	Primary	3,701 (41.7%)	603 (29.1%)	868 (31.9%)	
	Secondary	1,088 (12.2%)	78 (3.8%)	163 (6%)	
	Higher	507 (5.7%)	37 (1.8%)	60 (2.2%)	
Religion	Orthodox	3,685 (41.5%)	600 (29%)	1,192 (43.8%)	<0.001
	Catholic	47 (0.5%)	12 (0.6%)	11 (0.4%)	
	Protestant	2,435 (27.4%)	594 (28.7%)	771 (28.3%)	
	Muslim	2,619 (29.5%)	814 (39.3%)	732 (26.9%)	
	Others	98 (1.1%)	49 (2.4%)	17 (0.6%)	
Wealth index	Poor	3,052 (34.3%)	948 (45.8%)	973 (35.7%)	<0.001
	Middle	1,671 (18.8%)	414 (20%)	559 (20.5%)	
	Rich	4,162 (46.8%)	707 (34.2%)	1,192 (43.8%)	
Survival status of the previous child	No	282 (4.8%)	136 (6.6%)	94 (3.5%)	<0.001
	Yes	5,573 (95.2%)	1,933 (93.4%)	2,629 (96.5%)	
Current contraceptive method	Not using	6,329 (71.2%)	1,373 (66.4%)	1,618 (59.4%)	<0.001
	Using	2,556 (28.8%)	696 (33.6%)	1,105 (40.6%)	
Breastfeeding status	No	1,092 (32.4%)	400 (36.5%)	454 (29.9%)	<0.001
	Yes	2,275 (67.6%)	696 (63.5%)	1,066 (70.1%)	
Marital status	Single	2,580 (29%)	49 (2.4%)	68 (2.5%)	0.003
	Married	5,743 (64.6%)	1,845 (89.2%)	2,447 (89.9%)	
	Widowed	185 (2.1%)	94 (4.5%)	80 (2.9%)	
	Divorced	377 (4.2%)	80 (3.9%)	128 (4.7%)	

### Spatial autocorrelation of SBIs of women in Ethiopia

**Figure 2:**
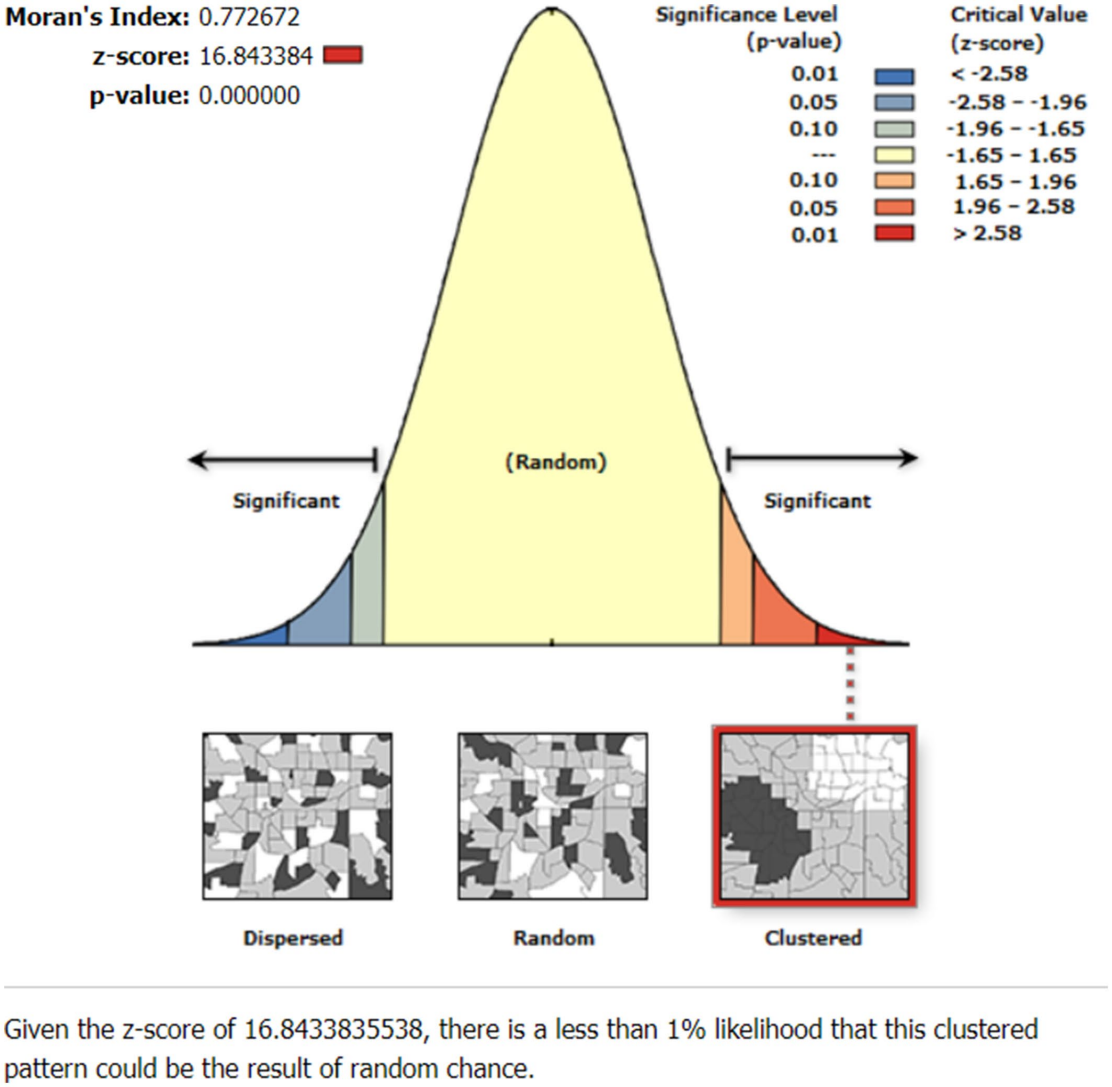
Spatial autocorrelation of short birth interval (SBI) in Ethiopia, EMDHS 2019.

The SBIs were spatially clustered in Ethiopian administrative zones with Global Moran’s *I* = 0.773 and *p* < 0.001 (Figure 2). The area of study had significant rates of SBIs, as indicated by the clustered patterns on the right sides. With a Z-score of 16.84, it is less than 1% that this clustered pattern is a result of random chance.

### Spatial distribution of short birth interval

**Figure 3:**
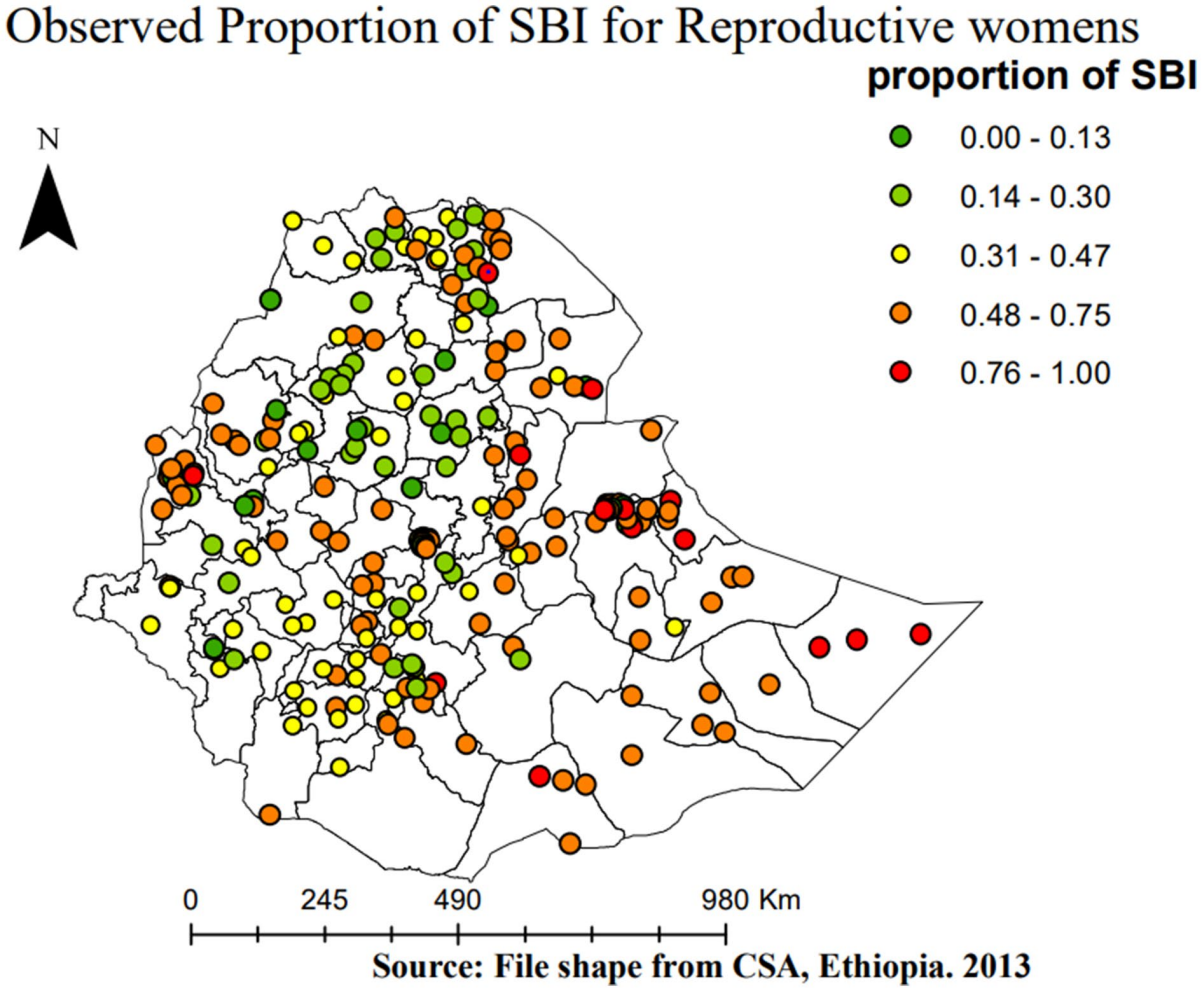
Spatial distribution of SBI across Ethiopian administrative zones.

Figure 3 shows the spatial distribution of the observed proportion of SBIs in Ethiopia across each enumeration area in EMDHS 2019, where high and low proportions of SBIs were represented by red and green colors, respectively.

### Hot spot (Getis-Ord Gi*) analysis of short birth interval

**Figure 4:**
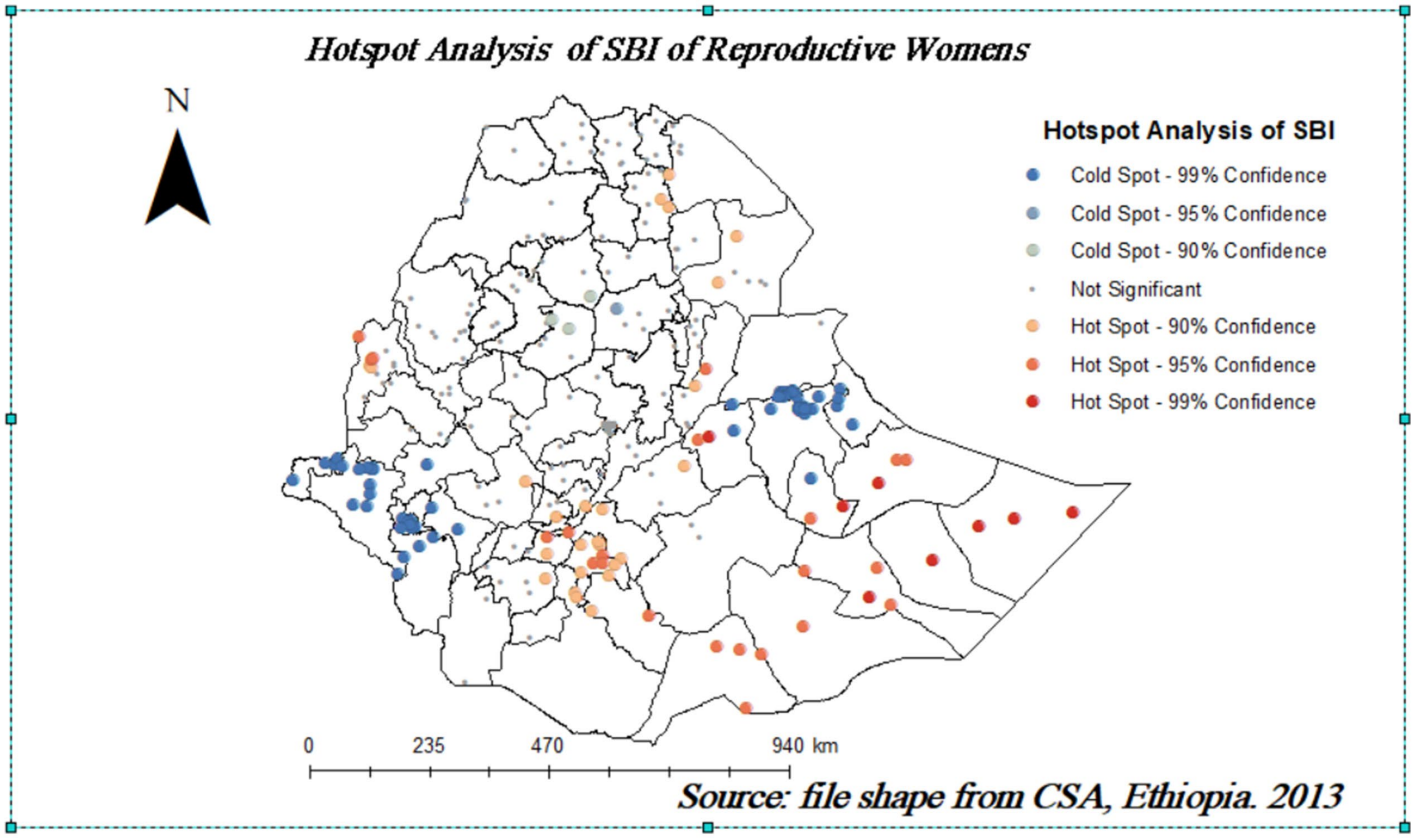
Hot spot analysis of SBI in Ethiopia’s administrative zones.

Figure 4 displays the hot spot areas of SBIs of reproductive women in Ethiopia. There are significant hot and cold spots in SBIs in Ethiopia, according to the Local Getis-Ord Gi* statistics. A point with a red color indicates significant hot spot areas of SBIs. Accordingly, Jarar, Doolo, Shabelle, Afder, Liben, Korahe, Nogob, West Harerge, Guji, Sidama, and Assosa were the hot spot areas of SBIs. Whereas the cold spot areas were Fafan, East Harerge, Ilubabur, Bench Maji, Keffa, Agnuak, and Nuer zones (Figure 4).

### Spatial determinates of short birth interval in the MEDHS data

First of all, the candidate predictor variables were fitted to the OLS regression model. This model satisfied every OLS assumption. The joint Wald statistics showed significance for the whole model (𝑝 < 0.001), and the predictor variables’ coefficient significance was shown by robust probabilities (𝑝 < 0.001). Additionally, multicollinearity was assessed using the coefficient of variance inflation factor, and there were no redundancy issues in the predictor variables (VIF < 7.5). The adjusted R2 = 0.43 indicates that 43% of the variation in SBIs was explained by the model. Therefore, the spatial determinates of hot spot areas of SBIs were illiterate women, women who had not breastfed, and orthodox followers (Table 2).

**Table 2:** Summary of spatial OLS results of SBI in Ethiopia, mini-Ethiopia demographic, and health survey (EMDHS) 2019.

Variable	Coefficient	Std Error	t-Statistic	Probability	Robus (SE)	Robust_t statistics	Robust_Probability	VIF
Intercept	0.052520	0.092744	0.566292	0.571635	0.071251	0.737116	0.461634	–
PILLTERATE	0.265947	0.054362	4.892121	0.000002∗	0.073742	3.606462	$0.000376^{*}$	1.993601
PNOTBRE	0.420776	0.050933	8.261432	$0.000000^{*}$	0.062438	6.739129	$0.000000^{*}$	1.157890
PORTHO	−0.078091	0.029927	−2.609384	0.009530∗	0.026500	−2.946882	0.003474∗	1.216877

*Significant at 5% level of significance.

Considering the fact that OLS analysis identified SBI hot spot area predictors, it makes the assumption that, across the study area, the relationships between each predictor variable and SBIs are stationary. However, this assumption is violated by the significant Koenker (BP) statistic (𝑝 < 0.001). This problem is better managed by a GWR model or a local model if stationarity is violated. A GWR model was therefore fitted to have a reliable estimator. In this model, the adjusted R2 value obtained from OLS increased from 0.43 in Table 2 to 0.46 using GWR (Table 3). This was further supported and taken into account by the modified AIC, for which the GWR provided a smaller value (AICc = −108.26; Table 3) compared to the OLS model (AICc = −95.303; Table 2). Thus, the GWR model was determined to be better.

**Table 3:** Geographically weighted regression (GWR) of SBI in Ethiopia, EMDHS 2019.

Predictor variables	Illiterate women, women who do not breastfeed, orthodox follower women
Effective number	25.005
Sigma	0.1976
Akaike information criteria (AICc)	−108.26
Residual square	10.936
Multiple R-square	0.4992
Adjusted R-square	0.4562

### OLS diagnostics

**Table:** 

Number of observations	305	Akaike’s information criterion (AICc)	−95.303
Multiple R-Squared	0.450085	Adjusted R-squared	0.430140
Joint F-Statistic	23.9490890	Prob(>F),(11,293) degrees of freedom:	$0.000000^{*}$
Joint Wald Statistic	673.47665995	Prob(> chi-squared), (11) degrees of freedom:	0.000000∗
Koenker (BP) Statistic	59.823809	Prob(> chi-squared), (11) degrees of freedom:	0.000000∗
Jarque–Bera Statistic	18.163598	Prob(> chi-squared), (2) degrees of freedom:	$0.000103^{*}$

**Figure 5:**
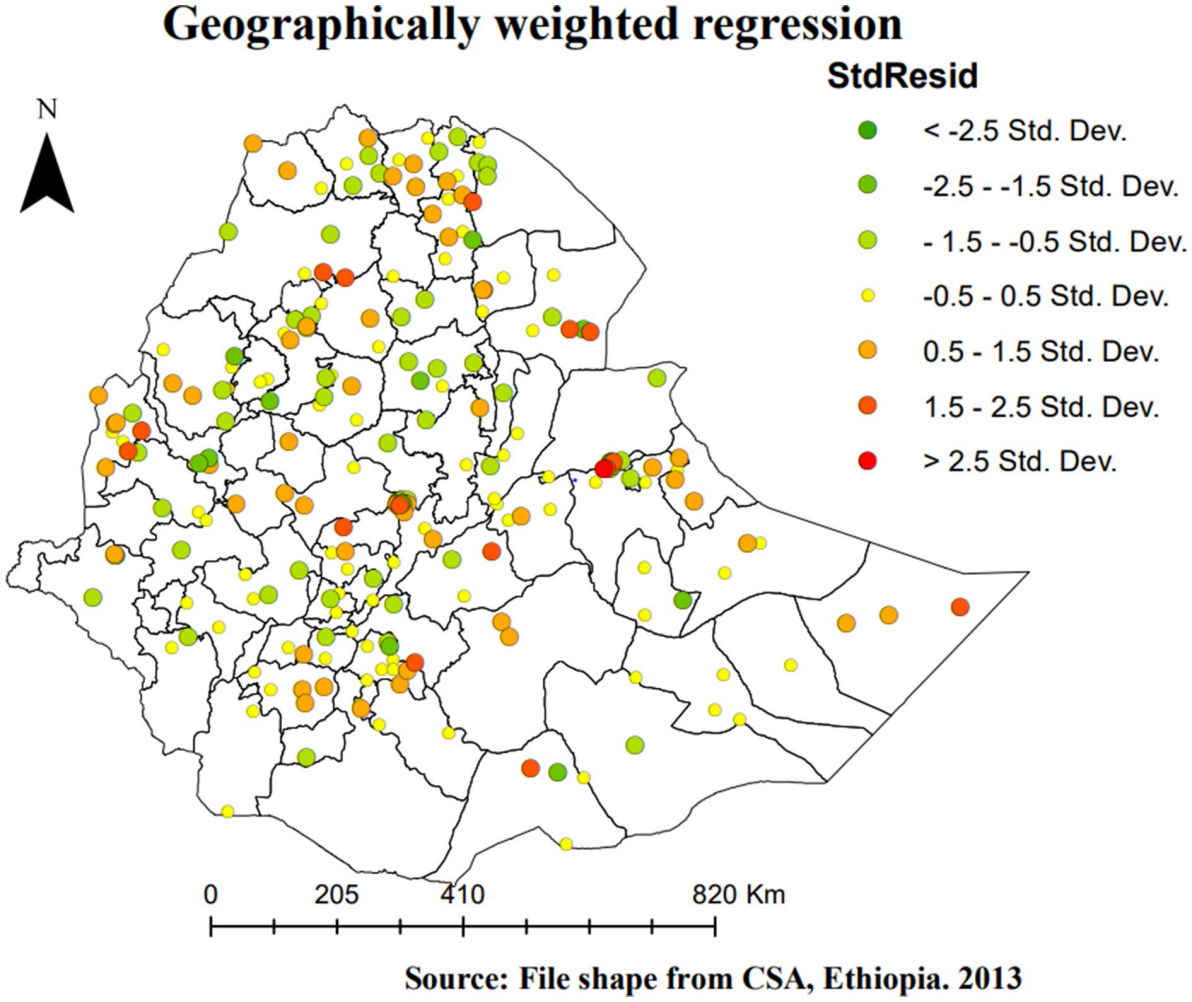
Geographically weighted regression StdResid of SBI in Ethiopia, EMDHS 2019.

Figure 5 shows the geographically weighted standardized residual of SBIs, which is the difference between the predicted and observed proportion of SBIs in the study area. The areas with red points indicate EAs where there is a relatively big difference between the observed and predicted (over-predicted) proportions of SBIs. Whereas the green points represent areas where SBIs were under-predicted.

**Figure 6:**
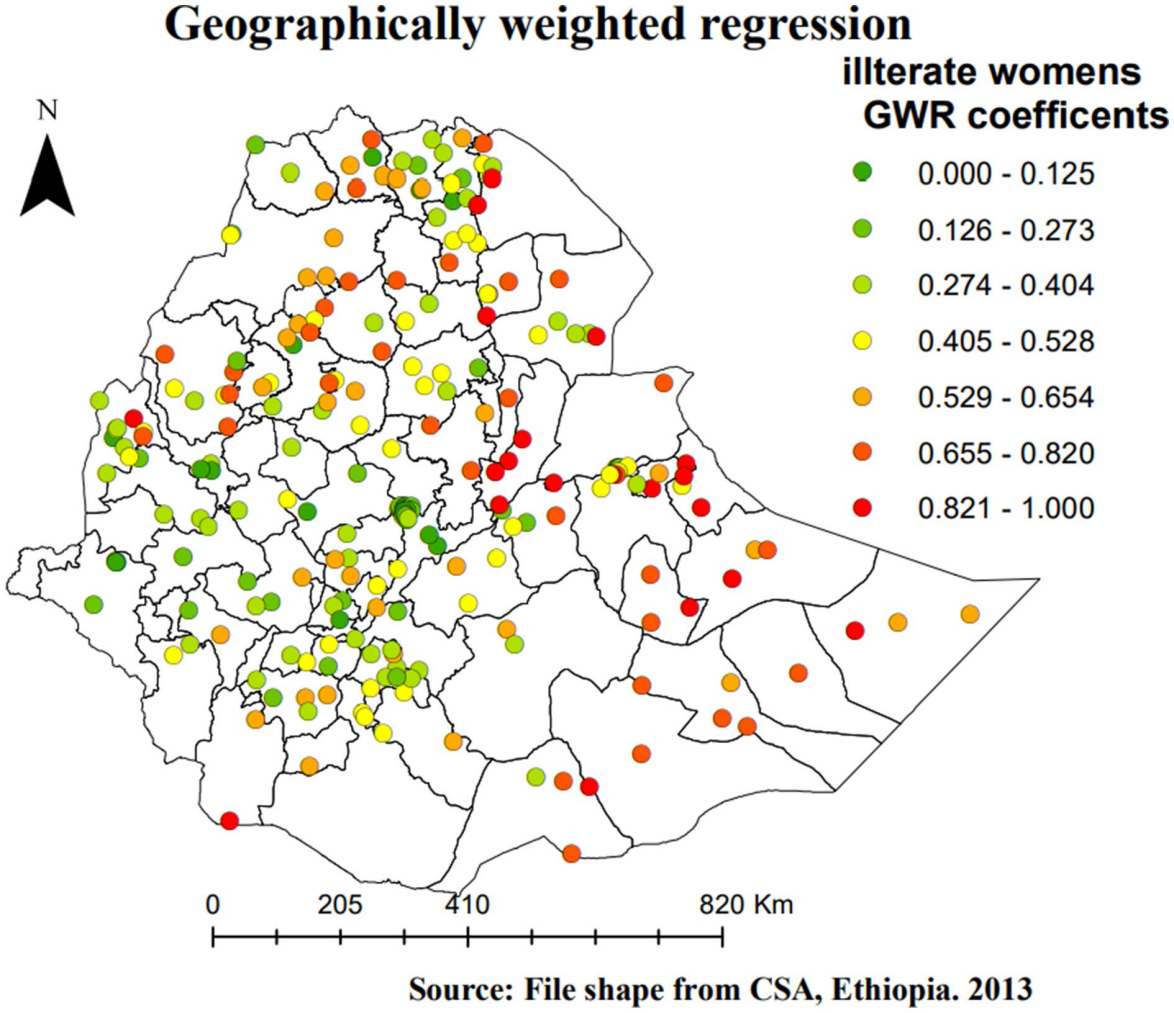
Geographically weighted regression coefficients c of illiterate women as a predictor of SBI in Ethiopia, EMDHS 2019.

**Figure 7:**
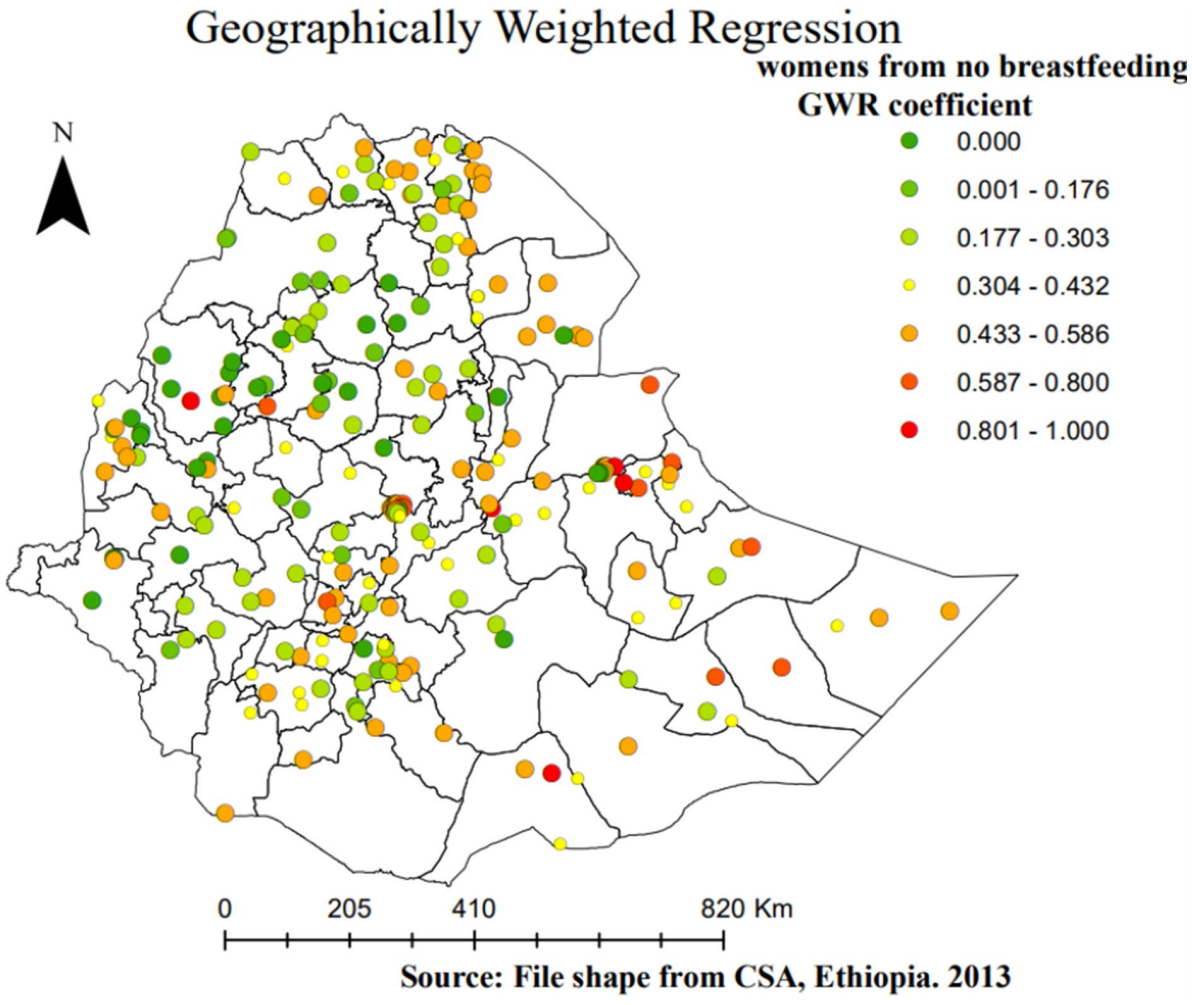
Geographically weighted regression coefficients of women who have no breastfeeding practice as a predictor of SBI in Ethiopia, EMDHS 2019.

**Figure 8:**
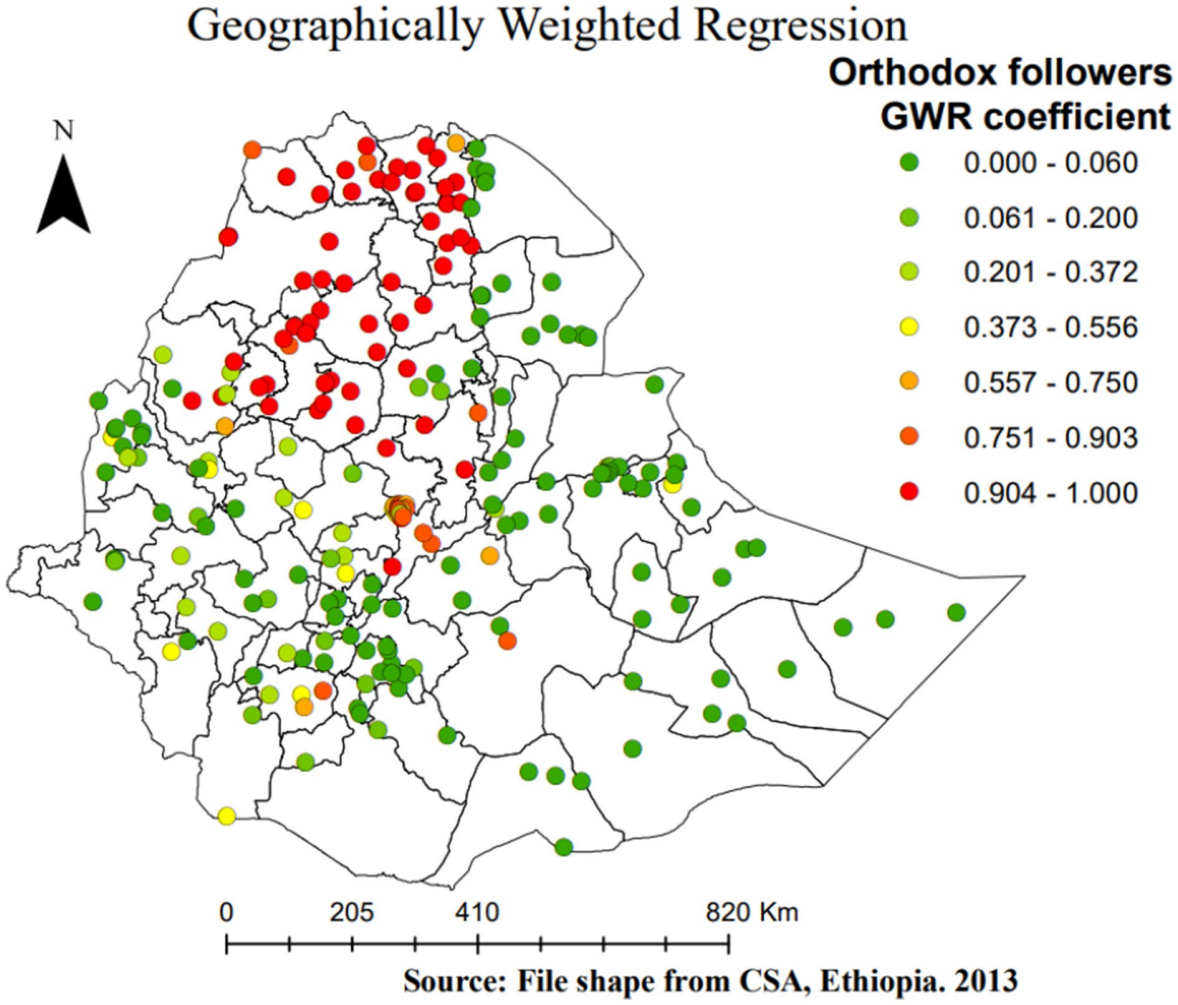
Geographically weighted regression coefficients of orthodox follower women as a predictor of SBI in Ethiopia, EMDHS 2019.

Figures 6–8 provide a list of areas in Ethiopia where the candidate predictor variables had a significant impact on SBIs. For instance, uneducated women had a positive relationship with SBIs, as the intervals of the GWR coefficients of SBIs (Figure 6) are all positive and range from 0 to 1. Thus, as the proportion of uneducated women increases, the proportion of SBIs also increases in a given enumeration area. EAs shaded in red (Figure 6) represent areas where SBIs were highly affected by uneducated women. This is observed in all zones of the Somali region, east Harerge, zone 1, zone 2, zone 3, and zone 4 in the Afar region, northwestern in Tigray, Metekel in the Benishangul-Gumz region, and North Gonder in the Amhara region (Figure 6).

However, the GWR coefficients (Figure 7) indicate the proportion of women in Ethiopia who had no breastfeeding practice, and SBIs have a positive relationship. The red-shaded points represent no breastfeeding practice as a strong positive predictor of SBIs, which ranges from 0 to 1. Thus, as the proportion of women with no breastfeeding practice increases, the proportion of SBIs also increases in a given enumeration area. This circumstance was observed somewhat around all zones of the Somali region, zone 1, zone 2, zone 3, and zone 4 in the Afar region, north Shewa in the Amhara region, central and western Tigray, Guji in the Oromia region, Hadiya in SNNPR, and Asossa (Benishangul-Gumz) region (Figure 7).

The other important spatial predictor of SBIs is the religion (being orthodox) of women, which has a positive relation with the percentage of SBIs (positive GWR regression coefficients). Thus, as the proportion of orthodox women in a given EA increases, the proportion of SBIs increases. Areas shaded in red indicate a high increase in SBI as the proportion of orthodox women increases. This is observed in all zones of Tigray, North and South Gonder, North Wollo, Waghimra, West and East Gojam, North Shewa, Awi (Agew) in the Amhara region, and East Shewa in the Oromia region (Figure 8).

## Discussion

Using several spatial analytical techniques, this study explored the spatial clustering and spatial causes of Ethiopian women’s SBIs. This study aimed to evaluate the spatial distribution and spatial factors of SBIs in Ethiopia using GWR. The descriptive analysis concluded that over 43.2% of women had SBIs. SBIs were clustered (Moran’s *I* = 0.773 and *p* < 0.001) in Ethiopia. This result was in line with the results of several studies from Ethiopia (2, 10, 20) and Bangladesh (21). SBI hotspots were also found in Jarar, Doolo, Shabelle, Afder, Liben, Korahe, Nogob, West Harerge, Guji, Sidama, and Assosa zones of Ethiopia. This claim is consistent with the findings of a study conducted in Ethiopia (22).

This study also found a relatively high prevalence of SBIs in the Afar region and Somali region, where a majority of SBI hot spots are located. These divisional variations in SBI hot spots are due to the division-level variations in sociodemographic and cultural characteristics of women and their partners and their perceptions regarding the desired number of children. Women who had no education, women who did not breastfeed, and women who were orthodox followers were factors in SBI hot spots. The three powerful predictors of SBI in Ethiopia’s administrative zones, according to their GWR coefficients. All three strong predictors range from 0 to 1 for women. This finding is in line with the study conducted in Ethiopia (9). In addition to the factors found in this study, the observed geographic variability of SBIs was associated with women who did not breastfeed in the Somali and Afar regions. It has long been recognized that women who breastfeed their children for a longer duration have a longer birth interval. This concept is confirmed by earlier research findings for hot spots for women who did not breastfeed in these regions. This could be caused by many things, such as gaps in media accessibility, knowledge of the best time to begin pregnancy, and the accessibility of medical resources (23). Additionally, variations in socioeconomic, cultural, and demographic aspects among those administrative zones may account for the variation in SBI hot spots. The majority of people in the Afar and Somali regions are pastoralists, who live a lifestyle characterized by seasonal migration (20). Access to health services and information is restricted for residents in certain places. Pastoralist communities are portable, but they also live in highly traditional environments and strongly follow cultural and religious norms (24). For example, research has indicated that women living in pastoralist societies have lower rates of high breastfeeding, which in turn affects the gap between pregnancies (24).

The GWR analysis showed a positive relationship between SBIs and women who had no breastfeeding status this study is consistent with a study by Regasa in Ethiopia (2). The current study also identified a spatially positive association between orthodox religion followers and SBIs among mothers in Ethiopia. This could be due to the non-use of contraceptive methods among orthodox followers, which is similar to the findings in Ethiopia. In any case, a few Muslims and Orthodox perceive it as a concept that is completely against the principles of Christianity and Islam. Participants who had never utilized contraceptive methods prior to the conception of the last child were more likely to be at risk of having SBIs compared to their counterparts (25, 26). There is some evidence to suggest that religious beliefs and values may influence individuals’ attitudes and behaviors regarding contraception and SBIs. For example, some religious teachings discourage the use of contraception, which may contribute to higher SBIs. However, it is important to recognize that there is a great deal of diversity within religious communities, and not all individuals or groups within a particular religion share the same beliefs and practices (27).

Not attending formal education by women had a positive relationship with SBIs. As the proportion of women who had not attended formal education increased, the occurrence of SBIs in the zones of the Somali region, zones of the Afar region, and some zones of the Tigray region increased (28). Additionally, women who did not pursue formal education were positively correlated with hot spots of SBIs in the far east of the Harerge zone in the Somali region. According to the published research, the Somali region has the largest percentage of uneducated women (75.0%), whereas Addis Ababa has the lowest percentage (9.0%) (12). By influencing women’s healthcare-seeking behaviors, such as their use of family planning services, education may have an impact on the spacing between pregnancies. Higher-educated women are also more likely to be more aware of health issues, to know more about the available services, to be better able to pay for medical care, and to have more autonomy when it comes to making decisions about their health, including family planning (29, 30).

In this investigation, it was found that different predictors of SBIs (women with no education, women with no breastfeeding status, and orthodox religion-following women) act more/less strongly across administrative zones. For example, women who did not attend formal education and women who did not breastfeed had a strong positive relationship with SBI hot spots in the Somali region, while they had a weak positive relationship with SBIs in the Amhara and Tigray regions. This may be due to differences in the accessibility and availability of various family planning services, as well as differences in socioeconomic status and culture throughout the nation’s administrative areas.

## Strengths and limitations

This study used a GWR model and the EDHS national datasets to examine the spatial effects of SBIs to identify most hot spot areas, which is considered a strength. The retrospective data collection procedure and the removal of several important variables that affect SBIs from the mini-2019 EDHS datasets resulted in bias in this analysis, which is considered a limitation of this study. In addition, the mini-EDHS data have certain limitations, especially regarding variable selection and biases inherent in self-reported data. While the mini-DHS data cover key demographic and health indicators, the primary limitation is the restricted set of variables collected. It may lack certain variables that could provide a more comprehensive understanding of the population under study. Second, respondents may provide inaccurate information due to memory lapses, social desirability bias, or misunderstanding of the questions. This can lead to inaccuracies and affect the reliability of the data.

## Conclusion

Overall, the study revealed geographic variations in Ethiopian women’s SBIs, with hot spots of SBIs identified in specific zones. Hot spots with SBIs were found in Jarar, Doolo, Shabelle, Afder, Liben, Korahe, Nogob, West Harerge, Guji, Sidama, and Assosa zones. Factors such as low education levels, lack of breastfeeding practices, and orthodox religious followers were associated with an increased risk of SBIs in these areas. Decision-makers at the national and regional levels are encouraged to prioritize the provision of family planning services in these hot spot communities. Engaging religious leaders is an effective strategy for promoting modern family planning. Community-based programs focusing on women’s education and empowerment can also play a significant role in reducing the impact of SBIs. Additionally, future research should consider integrating new data sources, such as socioeconomic status, access to healthcare, cultural norms, and community-level characteristics, to gain a more comprehensive understanding of the determinants of SBIs. Moreover, future studies should consider integrating a mixed-methods approach, combining quantitative and qualitative data, to capture the complex interplay of sociocultural factors influencing SBIs.

## Data availability statement

The original contributions presented in the study are included in the article/supplementary material, further inquiries can be directed to the corresponding author.

## Ethics statement

Ethical approval was not required for the studies involving humans because the data that we used is secondary. The studies were conducted in accordance with the local legislation and institutional requirements. Written informed consent for participation was not required from the participants or the participants’ legal guardians/next of kin in accordance with the national legislation and institutional requirements because the data that we used is secondary.

## Author contributions

GA: Conceptualization, Data curation, Formal analysis, Investigation, Methodology, Software, Supervision, Validation, Visualization, Writing – original draft, Writing – review & editing. AM: Methodology, Supervision, Validation, Writing – review & editing. NM: Methodology, Writing – review & editing. SM: Methodology, Writing – review & editing. HB: Methodology, Writing – review & editing. KA: Methodology, Writing – review & editing.

## Footnotes

^1^https://dhsprogram.com/
